# *Mycobacterium tuberculosis* ferritin: a suitable workhorse protein for cryo-EM development

**DOI:** 10.1107/S2059798321007233

**Published:** 2021-07-29

**Authors:** Abril Gijsbers, Yue Zhang, Ye Gao, Peter J. Peters, Raimond B. G. Ravelli

**Affiliations:** a Maastricht Multimodal Molecular Imaging Institute, Division of Nanoscopy, Maastricht University, Universiteitssingel 50, 6229 ER Maastricht, The Netherlands

**Keywords:** cryo-EM, ferritin, *Mycobacterium tuberculosis*, single-particle analysis, expression and purification protocols

## Abstract

The use of cryo-EM continues to expand worldwide and calls for good-quality standard proteins with simple protocols for their production. Here, a straightforward expression and purification protocol is presented that provides an apoferritin, bacterioferritin B (BfrB), from *Mycobacterium tuberculosis* with high yield and purity. A 2.12 Å resolution cryo-EM structure of BfrB is reported, showing the typical cage-like oligomer constituting of 24 monomers related by 432 symmetry. However, it also contains a unique C-terminal extension (164–181), which loops into the cage region of the shell and provides extra stability to the protein. Part of this region was ambiguous in previous crystal structures but could be built within the cryo-EM map. These findings and this protocol could serve the growing cryo-EM community in characterizing and pushing the limits of their electron microscopes and workflows.

## Abstract 2

A simple expression and purification protocol is presented which produces a high yield and good micrographs of the apoferritin bacterioferritin B (BfrB) from *Mycobacterium tuberculosis*. Its 2.12 Å resolution cryo-EM structure is presented, revealing a unique C-terminal extension (164–181), and it is discussed how BfrB could serve the growing cryo-EM community in characterizing and pushing the limits of their electron microscopes and workflows.

## 1. Introduction   

Single-particle cryogenic electron microscopy (cryo-EM) has become an indispensable tool for structural biology. The combination of direct electron detectors, motion correction (Scheres, 2014 ▸; Li *et al.*, 2013 ▸), high-end electron microscopes and advanced imaging-processing algorithms allowed the ‘resolution revolution’ (Kühlbrandt, 2014 ▸), since which an increasing number of single-particle analysis (SPA) structures with resolutions below 2 Å have been determined (Zivanov *et al.*, 2018 ▸; Bartesaghi *et al.*, 2018 ▸; Hamaguchi *et al.*, 2019 ▸; Tan *et al.*, 2018 ▸). Hardware improvements such as monochromators, spherical aberration (C_S_) correctors, energy filters and a new generation of direct electron detectors have improved the signal-to-noise ratio even further and have brought SPA to real atomic resolution (Nakane *et al.*, 2020 ▸; Yip *et al.*, 2020 ▸). The success of SPA has attracted many new scientists into the field of cryo-EM and has led to an impressive growth in the community as well as in the number of instruments that can deliver high-resolution cryo-EM structures. Ferritin is often used as a benchmark to commission and validate these machines, as it is relatively straightforward to obtain good-resolution data sets for its 24-mers. In fact, as of March 2021, 11 of the 25 SPA structures deposited in the Protein Data Bank (PDB) with a resolution of better than 2 Å are of ferritins from different organisms. Ferritin has thus become a gold standard for cryo-electron microscopists to evaluate their setups and to push the limits of sample preparation (Ravelli *et al.*, 2020 ▸; Russo & Passmore, 2014 ▸; Jain *et al.*, 2012 ▸; Carragher *et al.*, 2019 ▸), imaging (Yip *et al.*, 2020 ▸; Nakane *et al.*, 2020 ▸) and data processing (Zhang *et al.*, 2020 ▸; Zivanov *et al.*, 2018 ▸; Punjani *et al.*, 2017 ▸).

Ferritins are protein complexes that are involved in iron homeostasis and DNA repair by storing iron in cage-like structures. This protein complex is formed by 12 or 24 subunits with tetrahedral 23-symmetric or octahedral 432-symmetric arrangements, depending on the subfamily to which it belongs. There are three subfamilies of ferritins: iron-containing ferritins and heme-containing bacterioferritins involved in iron storage, and DNA-binding protein in starved cells (Dps), which uses iron to protect DNA from free radical-mediated damage (Andrews, 2010 ▸; Arosio *et al.*, 2009 ▸). Iron is a trace element that is vital for all living organisms, and although it is the second most common metal on Earth, it is not always bio­available: for this reason, organisms compete for the iron in the environment (Expert, 2012 ▸). The acquisition of iron is considered to be a key step in the development of any pathogen, and so host cells have developed mechanisms to sequester the metal from infecting bacteria in an attempt to defend themselves (Ratledge & Dover, 2000 ▸). One such pathogen is *Mycobacterium tuberculosis*, the causative agent of human tuberculosis.

In this study, a simple high-yield purification protocol is presented for bacterioferritin B (BrfB) from *M. tuberculosis*, together with its high-resolution cryo-EM structure. Previously, only lower resolution crystal structures have been reported. Our structure shows extra residues at both termini. The C-terminal tail of BrfB, which is a target peptide for encapsulation (Contreras *et al.*, 2014 ▸) and which plays a role in ferroxidase activity and iron release in addition to providing stability to the protein (Khare *et al.*, 2011 ▸), loops into the cage region of the complex prior to folding back via the threefold channel to the B-pore. Two conformations of the C-terminal residue His175 were observed near the B-pore, suggesting a role of this residue in iron exchange. BfrB could serve the structural community in testing their expanding fleet of equipment as well as aiding in a better understanding of iron-storage proteins, which are essential for the survival and the progression of important human pathogens such as *M. tuberculosis*.

## 2. Materials and methods   

### 2.1. Expression and purification of BfrB   

The codon-optimized gene for *M. tuberculosis* BfrB was cloned into a modified pRSET backbone (Eurofins Genomics) using NdeI and HindIII restriction sites for overexpression in *Escherichia coli* C41(DE3) cells. The expression and purification protocol was adapted from Khare *et al.* (2011 ▸) and Parida *et al.* (2020 ▸). In summary, a primary culture of lysogeny broth (LB) medium supplemented with 100 µg ml^−1^ ampicillin was prepared from a single colony at 37°C and 200 rev min^−1^ overnight and was then used to inoculate 500 ml fresh LB medium with a 1:1000 dilution of the primary culture in the same conditions until an optical density (OD_600_) of 0.6 was reached. Protein overexpression was induced by adding isopropyl β-d-1-thiogalactopyranoside (IPTG) to a final concentration of 1 m*M* for 3 h without changing the culture conditions. The cells were harvested by centrifugation and were stored at −20°C until further use.

The bacterial cells were resuspended in 20 m*M* Tris–HCl pH 8.0, 300 m*M* NaCl supplemented with 2 U ml^−1^ benzonase (Sigma–Aldrich), EDTA-free protease inhibitor (Sigma–Aldrich) and 2 m*M* β-mercaptoethanol (BME). The cell suspension was lysed by sonication. Cellular debris was removed by centrifugation at 30 000*g* for 30 min. The supernatant was subjected to a saturation of 20% ammonium sulfate and incubated at 5°C for 1 h with constant shaking before centrifugation at 15 000*g* for 20 min. The pellet was resuspended in 20 m*M* Tris–HCl pH 8.0, 150 m*M* NaCl and centrifuged at 10 000*g* for 5 min to remove precipitants. The sample was further purified by size-exclusion chromatography on a Superdex 200 Increase 10/30 column (GE Healthcare). Fractions were collected and the protein purity was evaluated by denaturing polyacrylamide gel electrophoresis. Fractions containing BfrB were pooled and were stored at −80°C until further use. The final yield was >100 mg per litre of culture.

### 2.2. Cryo-electron microscopy sample preparation, data acquisition and image processing   

**Figure 1:**
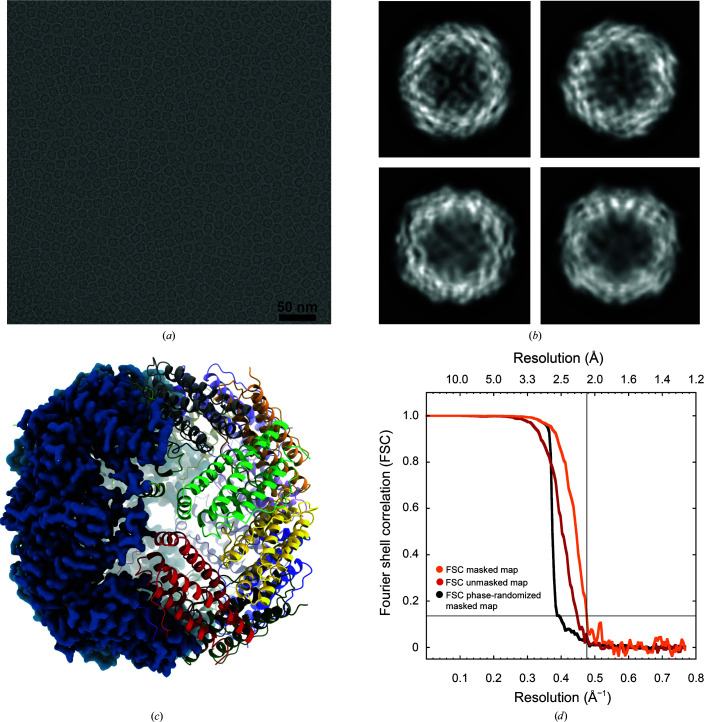
Single-particle analysis of BfrB. (*a*) A micrograph of a highly concentrated (80 mg ml^−1^) BfrB sample in vitreous ice collected on a Falcon 3 at 200 kV. (*b*) 2D class averages; the size of the shown box is 150 Å. (*c*) 3D reconstruction from 163 568 particles at 2.12 Å resolution collected on a K3 at 300 kV. (*d*) Gold-standard Fourier shell correlation (FSC) before (red line) and after (orange line) masking and the phase-randomized FSC (black line).

Purified BfrB was first used at a concentration of 11 mg ml^−1^ (as calculated with the Pierce BCA Protein Assay Kit). A volume of 2.5 µl was applied onto glow-discharged UltrAuFoil Au300 R1.2/1.3 grids (Quantifoil). Excess liquid was removed by blotting for 3 s (blot force 5) using filter paper followed by plunge freezing in liquid ethane using an FEI Vitrobot Mark IV operated under 100% humidity at 4°C. Cryo-EM single-particle data were collected on a Titan Krios at 300 kV with a BioQuantum K3 Imaging Filter with a 20 eV post-column energy filter. The detector was utilized in super-resolution counting mode at a nominal magnification of 130 000×. Table 1 ▸ shows the statistics of the data set. Data were processed using the *RELION* pipeline (Scheres, 2012 ▸). Movie stacks were corrected for drift (5 × 5 patches) and dose-weighted using *MotionCor*2 (Zheng *et al.*, 2017 ▸). The local contrast transfer function (CTF) parameters were determined for the drift-corrected micrographs using *Gctf* (Zhang, 2016 ▸). A first set of 2D references were generated from manually picked particles in *RELION* (Scheres, 2012 ▸) and these were then used for subsequent automatic particle picking. Table 1 ▸ lists the number of particles in the final data set after particle picking, 2D classification and 3D classification. The latter was with *O* symmetry. Beam-tilt parameters, anisotropic magnification and local CTF parameters were refined and the particles were polished (Zivanov *et al.*, 2018 ▸). The resolution of the final full map, listed in Table 1 ▸ and shown in Fig. 1 ▸, was 2.12 Å using the gold-standard FSC = 0.143 criterion (Scheres & Chen, 2012 ▸). A *B*-factor plot according to Rosenthal & Henderson (2003 ▸) was calculated using random subsets of the data with variable numbers of particles (Supplementary Fig. S1).

Table 1 ▸ also includes the statistics of a data set collected in-house on a Tecnai Arctica microscope, operating at 200 kV, with a Falcon 3 camera operated in electron-counting mode (no energy filter). For this data set, we used a highly concentrated sample of 80 mg ml^−1^, which gave beautiful micrographs with densely packed monolayers of BfrB in the middle of the holes in the UltrAuFoil Au300 R1.2/1.3 grids (Fig. 1 ▸ *a*). This data set contained only a third of the number of micrographs compared with the K3 data set; however, since it was collected from a more concentrated sample with a larger pixel size (0.935 versus 0.651 Å), similar numbers of particles were obtained for both data sets. The resolution of the 200 kV Falcon 3 data set was 2.39 Å.

### 2.3. Structure determination and refinement   

We used PDB entry 3qd8 (Khare *et al.*, 2011 ▸) as a starting model in *Coot* (Emsley & Cowtan, 2004 ▸) for manual docking and building. The final model was refined against a sharpened cryo-EM map obtained by *LocSpiral* (Kaur *et al.*, 2021 ▸). The model was refined iteratively through rounds of manual adjustment in *Coot* (Emsley *et al.*, 2010 ▸), real-space refinement in *Phenix* (Afonine *et al.*, 2018 ▸) and structure validation using *MolProbity* (Williams *et al.*, 2018 ▸). The refined model has been deposited in the Protein Data Bank as PDB entry 7o6e and the maps have been deposited in the Electron Microscopy Data Bank as entry EMD-12738.

## 3. Results and discussion   

### 3.1. Mycobacterial ferritin structure   

**Figure 2:**
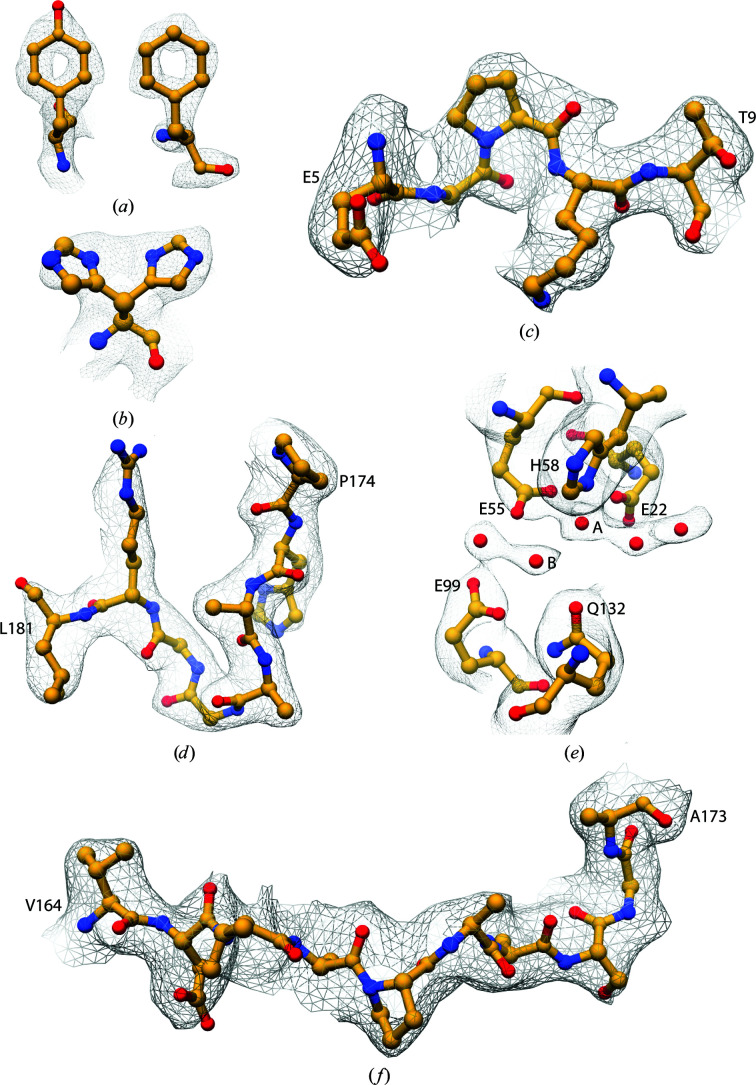
Representative regions of the density. (*a*) Post-processing map density for Phe23 and Tyr35. Local resolution scaled maps of (*b*) His175, (*c*) Glu5–Thr9 and (*d*) C_rigid_ (Pro174–Leu181). (*e*) A string of density blobs near the ferroxidase sites. (*f*) C_flex_ (Val164–Ala173). The EM density is shown as a grey mesh; the residue atoms are represented as a ball-and-stick model.

We studied the structure of BrfB by cryo-EM and obtained a 2.12 Å resolution density map based on the gold-standard FSC (Scheres & Chen, 2012 ▸) using 2518 micrographs with 163 568 particles. The high-quality data allowed the observation of secondary-structure features during 2D classification (Fig. 1 ▸). The clear density for side chains and holes in aromatic rings illustrates the quality of the EM map (Fig. 2 ▸). Our final model contained 1392 water molecules, whereas the BfrB model built from the 3 Å resolution X-ray crystallography map had 360 water molecules (Khare *et al.*, 2011 ▸) and the 1.15 Å resolution cryo-EM structure of human apoferritin had 4622 water molecules (Yip *et al.*, 2020 ▸).

Previous models of *M. tuberculosis* BfrB have been determined by X-ray crystallography (PDB entries 3qd8 at 3 Å resolution, 3oj5 at 2.85 Å resolution and 3uno at 2.5 Å resolution; Khare *et al.*, 2011 ▸; TB Structural Genomics Consortium, unpublished work). We built our model in an enhanced cryo-EM map, a *LocSpiral* map, calculated using algorithms based on spiral phase transformation (Fig. 2 ▸; Kaur *et al.*, 2021 ▸). Supplementary Fig. S3(*a*) shows a comparison between the *RELION* postprocess map and the *LocSpiral* sharpened map, revealing some extra features in the latter. To confirm these, we also calculated *LocOccupancy* maps, which estimate the density occupancy (Supplementary Fig. S3*b* ; Kaur *et al.*, 2021 ▸). The resolution of both maps was also estimated by comparing the FSC between the refined model and the map (Afonine *et al.*, 2018 ▸) at a cutoff value of 0.5 (Supplementary Fig. S2): this was 1.89 and 2.18 Å when compared with the *LocSpiral* and *RELION* post-processing maps, respectively (Table 1 ▸).

**Figure 3:**
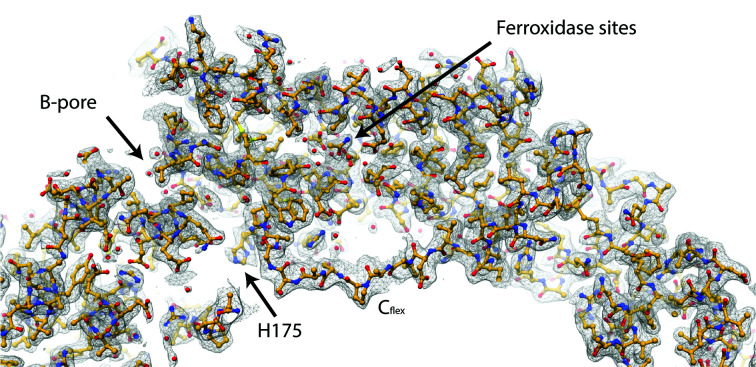
Density map and model for C_flex_, which extends into the interior of the cage and is located above the cavity between the B-pore and the ferroxidase centres.

At the N-terminus, we could add residues Glu5–Thr9, for which clear density was seen in the enhanced EM map (Fig. 2 ▸ *c*) but which were absent in our starting model (PDB entry 3qd8). The C-terminal region 164–181 consists of a flexible part (C_flex_, 164–173) and a rigid part (C_rigid_, 174–181), as described by Khare *et al.* (2011 ▸). C_flex_ is ill-defined within the X-ray maps. The C-terminal region has been shown to be important for protein stabilization and iron uptake (Khare *et al.*, 2011 ▸), and thus confident assignment of these residues could aid our understanding of BfrB function. The extension of the C-terminal end in *M. tuberculosis* is unusual for ferritin even compared with the heme-containing BfrA from the same organism. This extension has been shown to play an essential role in its function: BfrB exhibits a 3.5-fold reduction in the oxidation rate of iron(II) and a 20% reduction in the iron(III) release rate upon removal of the C-terminal end (Khare *et al.*, 2011 ▸). We found the extension of the C-terminal end to be located within the interior of the BfrB cage, which is remarkable as Mt-enc has been reported to encapsulate BfrB via this C-terminal extension (Contreras *et al.*, 2014 ▸). Our EM map displays well defined density for C_rigid_ (Fig. 2 ▸ *d*), residues 174–181, including a double conformation of residue His175 (Fig. 2 ▸ *b*). Similar to as in *S. coelicolor* bacterioferritin, Fe^2+^ enters BfrB from the B-pore and is converted to Fe^3+^ at the ferroxidase centres (Rui *et al.*, 2012 ▸; Jobichen *et al.*, 2021 ▸; Khare *et al.*, 2011 ▸). We hypothesize that the double conformation of His175 might be relevant to the iron exchange of the protein, as this residue is located in the interior of the B-pore and in the vicinity of a cavity (Fig. 3 ▸ and Supplementary Fig. S4). This cavity leads towards ferroxidase sites A (Glu22, Glu55 and His58) and B (Glu55, Glu99 and Glu135) where the ferrous iron is oxidized by molecular oxygen (Khare *et al.*, 2011 ▸; Parida *et al.*, 2020 ▸). Previously deposited X-ray diffraction electron-density maps did not place iron ions at these sites. The 2.5 Å resolution map for PDB entry 3uno only showed good density for the B site and weak density for the A site: water molecules were placed at both positions. The highly conserved Gln132 might favour iron binding at site B. The 3.0 Å resolution map for PDB entry 3qd8 showed some unmodelled density at the B site and no density for the A site. Finally, the 2.85 Å resolution map for PDB entry 3oj5 did not show clear density for either the A or the B site. In our EM map, a string consisting of five density blobs could be seen (Fig. 2 ▸ *e*). We placed water molecules here, as we lack experimental evidence for these water molecules being ions. Both the A and the B site have clear densities, where the A site is coordinated by Glu22 (2.55 Å), Glu55 (2.78 and 2.82 Å), His58 (2.51 Å) and neighbouring water molecules (2.44 and 2.65 Å). The B site is coordinated by Glu55 (2.77 Å), Glu99 (3.03 Å), Gln132 (2.55 Å) and another water molecule (2.55 Å). Glu135 has a different conformation compared with the X-ray map and does not coordinate to the A or B site. The coordination distances are longer then those most commonly found for iron ions according to *MetalPDB* (Putignano *et al.*, 2018 ▸): 2.031–2.236 Å for Fe–N and 2.077–2.414 Å for Fe–O. Another string of multiple water-like densities was found near Asp37, Pro42, Lys46 and Ser50.

Our map reveals density for C_flex_, which extends into the interior of the cage and is located above the cavity between the B-pore and the ferroxidase centres (Fig. 3 ▸). The density for C_flex_ is less defined than for other areas of the molecule (Fig. 2 ▸ *f* and Supplementary Fig. S3), which would reflect flexibility and a possible functional role of this (Teilum *et al.*, 2009 ▸). *LocSpiral* (Kaur *et al.*, 2021 ▸) helped to further improve this part of the density.

### 3.2. Suitable characteristics of a good standard protein for cryo-EM   

Ferritin has become more and more popular as a standard protein for testing and training purposes in microscopy laboratories. The good solubility and stability of most ferritins and their good intermediate size (12–13 nm diameter), combined with the 432 point-group symmetry, allows swift characterization of the performance of the microscope using a minimal number of micrographs. To date, the most commonly used samples are commercially available horse spleen ferritin and self-produced mouse ferritin (Wu *et al.*, 2020 ▸) and human ferritin (Yip *et al.*, 2020 ▸). However, the samples and protocols are not always optimal. The most affordable sample is horse spleen ferritin, but unfortunately it seldom provides the sample quality needed to push the resolution, possibly due to the presence of broken particles that introduce sample heterogeneity. Khare *et al.* (2011 ▸) compared the stability of ferritin subunits from different organisms and reported that horse spleen ferritin is one of the least stable, together with mouse ferritin. The highest resolution achieved by cryo-EM is on human ferritin (Yip *et al.*, 2020 ▸); however, the procedure described is an extensive protocol with more than ten steps that include the precipitation of nucleic acids, two ammonium sulfate precipitations, two sucrose gradients, two 24 h dialyses and ion-exchange chromatography. Although the final yield was not reported, it is expected that some protein will be lost in every step of the purification, resulting in high purity but low yield. The length and the use of so many different steps is inconvenient if we are aiming for a protocol that can be reproduced worldwide, as it will require infrastructure and expertise that might not be available everywhere. Previously, we have tried expression and purification protocols of constructs from different organisms, leading to yields that were, in our hands, not sufficient for the extensive and routine use of the proteins with classical grid-preparation techniques such as the Vitrobot.

**Figure 4:**
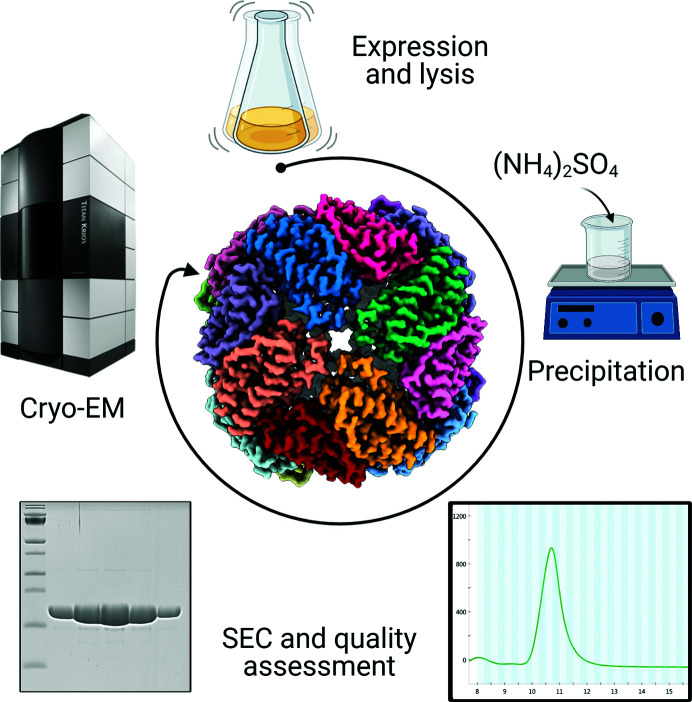
Simple purification workflow of the BfrB protein. After protein expression, *E. coli* cells are lysed by sonication and cellular debris is removed by centrifugation. The protein sample was precipitated by ammonium sulfate and resuspended for further purification by size-exclusion chromatography. A high yield of highly pure protein suitable for cryo-EM studies was obtained.

Here, we have provided an easy and cheap method to purify mycobacterial BfrB recombinantly expressed in the *E. coli* C41 strain. The expression and purification protocol was adapted from Khare *et al.* (2011 ▸) to increase the yield from ∼17.5 to >100 mg of protein per litre of culture. Given the good expression, we did not design a tag for the BfrB plasmid, thereby overcoming the need for a tag-removal step. One ammonium sulfate precipitation step followed by size-exclusion chromatography was sufficient to obtain samples with high levels of purity, as confirmed by SDS–PAGE (Fig. 4 ▸ and Supplementary Fig. S5) as well as cryo-EM (Fig. 1 ▸). A protein concentration of up to 115 mg ml^−1^ (calculated from the theoretical extinction coefficient) was achieved without any obvious precipitation, and no monomers were observed in the chromatogram, suggesting high solubility and stability of the purified BfrB oligomer. The 2.12 Å resolution structure presented here was obtained from one 11 mg ml^−1^ aliquot stored at −80°C, indicating that the protein is resistant to at least one freeze–thaw cycle. We prepared grids up to a concentration of 115 mg ml^−1^ and observed dense packing of non-overlapping BfrB particles at 80 mg ml^−1^ (Fig. 1 ▸ *a*), which is remarkably high but is reproducible with our local grid-preparation system. The *B*-factor plot according to Rosenthal & Henderson (2003 ▸) shows that we obtained resolutions of better then 4, 3 and 2.5 Å for 100, 1000 and 10 000 particles, respectively (Supplementary Fig. S1). Although the fitted *B* factor of 75 Å^2^ of our reported data set is too high to expect record-breaking resolutions, it is a perfectly adequate sample to obtain 2.5 Å resolution with modest microscopes and settings (Table 1 ▸). Whereas the rigid part of BrfB can serve in characterizing the performance of the microscope at regular times, the flexible C-terminus could also help to train users in some of the more advanced image data-processing SPA steps such as particle subtraction and focused classification. Plasmids are available upon request.

Over the last decade, there has been rapid development in every aspect of cryo-EM. Recent publications have proven that this technique has the potential to visualize macromolecules at the atomic level (Yip *et al.*, 2020 ▸; Nakane *et al.*, 2020 ▸). However, every protein has different physicochemical properties that makes it unique and that could challenge its structural elucidation by cryo-EM. For this reason, further improvements in cryo-EM are still necessary and thus a protein that serves as a ‘workhorse’ is needed to test them. BfrB could be one such protein.

## Supplementary Material

PDB reference: bacterioferritin B, 7o6e

EMDB reference: bacterioferritin B, EMD-12738

Supplemenatry Figures. DOI: 10.1107/S2059798321007233/vo5005sup1.pdf

## Figures and Tables

**Table 1:** Data-collection statistics for *M. tuberculosis* BfrB

Data collection		
Concentration (mg ml^−1^)	11	80
Grid type	Quantifoil UltrAuFoil 300 mesh R1.2/1.3	Quantifoil UltraAuFoil 300 mesh R1.2/1.3
Plunge freezer	Vitrobot	Vitrobot
Microscope	Krios	Tecnai Arctica
Voltage (kV)	300	200
Energy filter (eV)	20	None
Camera	K3	Falcon 3
Detector mode	Super-resolution counting	Electron counting
Nominal magnification (1000×)	130	110
Physical pixel size (Å)	0.6514	0.935
Exposure time (s)	1.3	46.33
Fluence (e^−^ Å^−2^)	40	41
Focus range (µm)	−0.4, −0.6, −0.7, −0.8, −0.9, −1.0, −1.2, −1.4	−0.75, −1.0, −1.25
Micrographs	2518	875
No. of fractions	50	50
Particles	163568	186025
Symmetry imposed	*O*	*O*
Average resolution (Å)	2.13	2.39
FSC threshold	0.143	0.143
Map-sharpening *B* factor (Å^2^)	−68	−109
Refinement		
Initial model used (PDB entry)	3qd8	
Model resolution against *LocSpiral* map (Å)	1.89	
FSC threshold	0.5	
Model resolution against *RELION* map (Å)	2.18	
FSC threshold	0.5	
Model composition of monomer		
Atoms	1471	
H atoms	0	
Protein residues	177	
Waters	58	
*B* factors (Å^2^)		
Protein	28.33	
Water	30.79	
R.m.s. deviations		
Bond lengths (Å)	0.017	
Bond angles (°)	1.324	
Correlation coefficients		
Mask	0.88	
Box	0.87	
Validation		
*MolProbity* score	1.20	
Clashscore	3.49	
Poor rotamers (%)	0	
Ramachandran plot		
Favoured (%)	97.71	
Allowed (%)	2.29	
Disallowed (%)	0	
