# Supplementary material for: Mycobacterium tuberculosis ferritin: a suitable workhorse protein for cryo-EM development
Source: Acta Crystallogr D Struct Biol. 2021 Jul 29;77(Pt 8):1077–83. doi: 10.1107/S2059798321007233 (PMC8329864; doi:10.1107/S2059798321007233)
Supplement: Supplementary file 1 [file d-77-01077-sup1.pdf]

STRUCTURAL BIOLOGY

Volume 77 (2021)

Supporting information for article:

Mycobacterium tuberculosis ferritin: a suitable workhorse protein for cryo-EM development

Abril Gijsbers, Yue Zhang, Ye Gao, Peter J. Peters and Raimond B. G. Ravelli

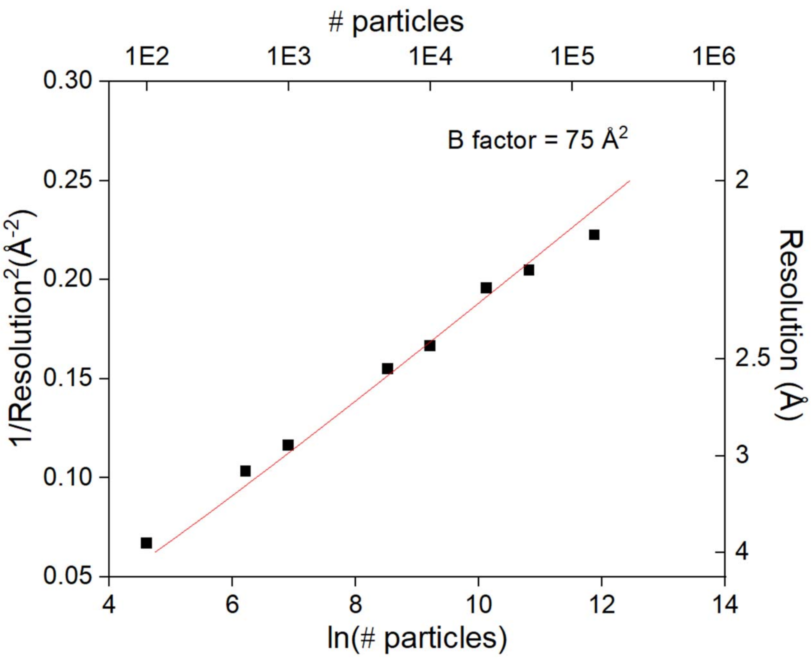

Figure S1 B-factor plot according to Rosenthal and Henderson (2003), where $1/d^2$, with d being the resolution of each refinement, is plotted against the natural logarithm of the number of particles in each subset. The fitted B-factor is 75 Å^2.

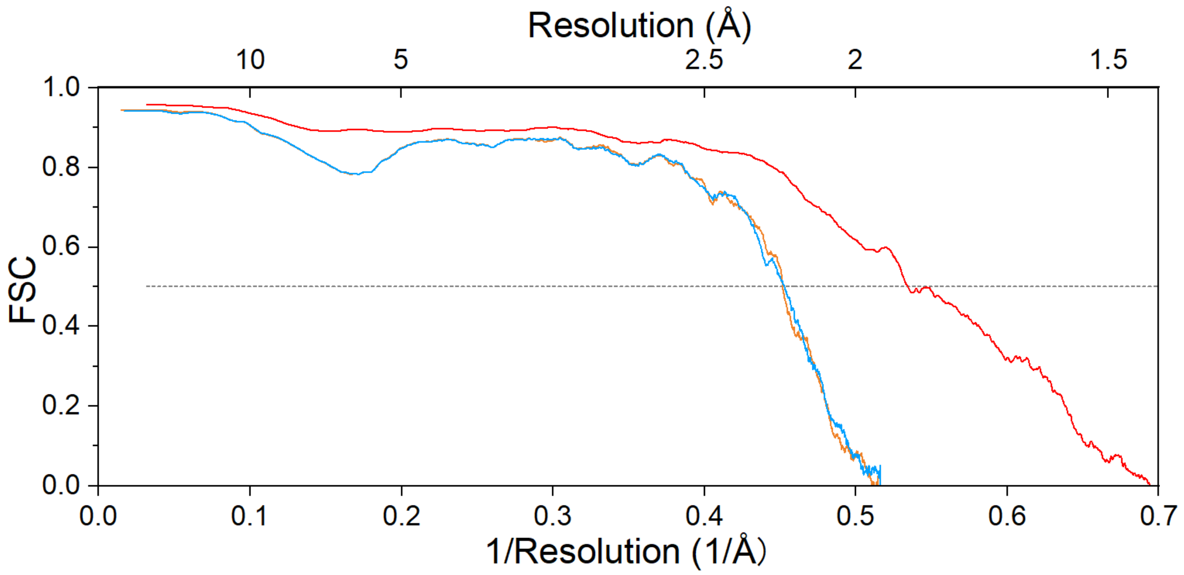

Figure S2 Fourier Shell Correlation (FSC) between map and model. The blue and orange lines are the FSC curves between model and both half maps. The red curve reports a resolution for the final map, according to the FSC (model) = 0.5 criterium, of 1.89 Å.

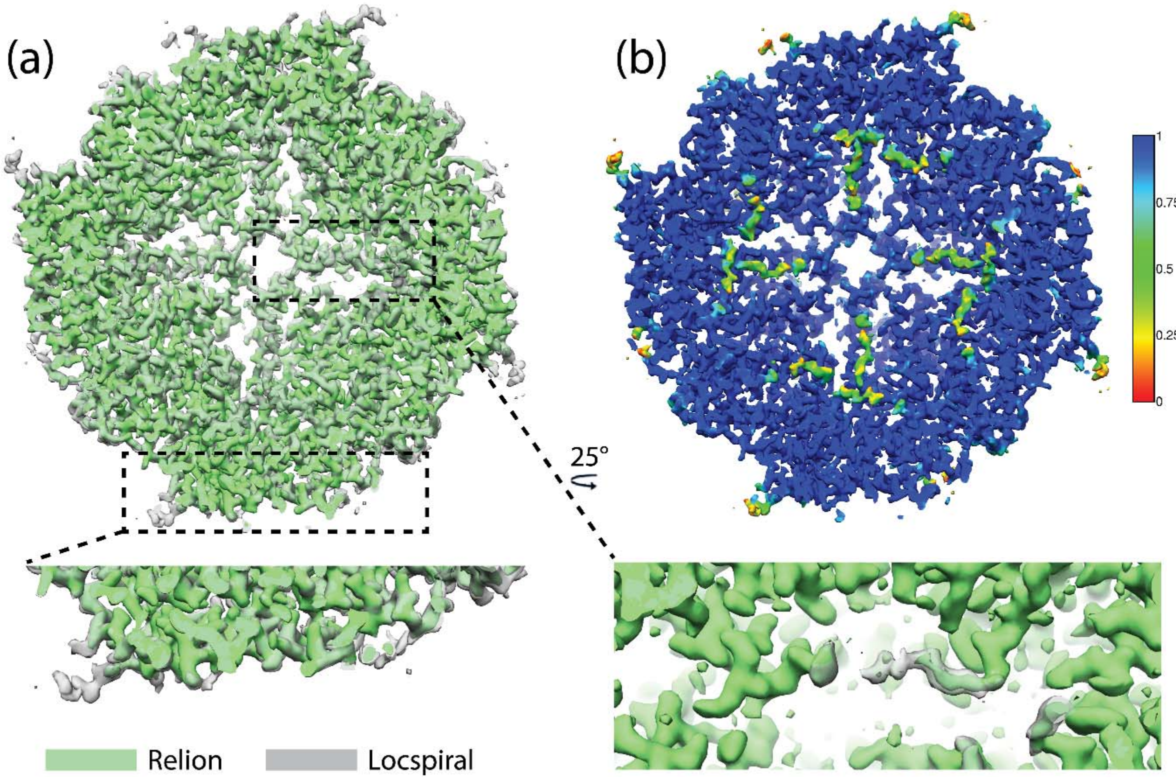

Figure S3 (a) The superimposed sharpened maps obtained by LocSpiral (grey colour) and Relion postprocessing (green colour). The figures below show zoomed view of the region indicated with the dashed rectangles. (b) The occupancy map of *Mtb* BfrB model obtained by LocOccupancy, calculated between 8 and 2.1 Å (Kaur *et al.*, 2021). The occupancy ranges between [0, 1], the occupancy of residues at N-term and Cflex have occupancy lower than 1.

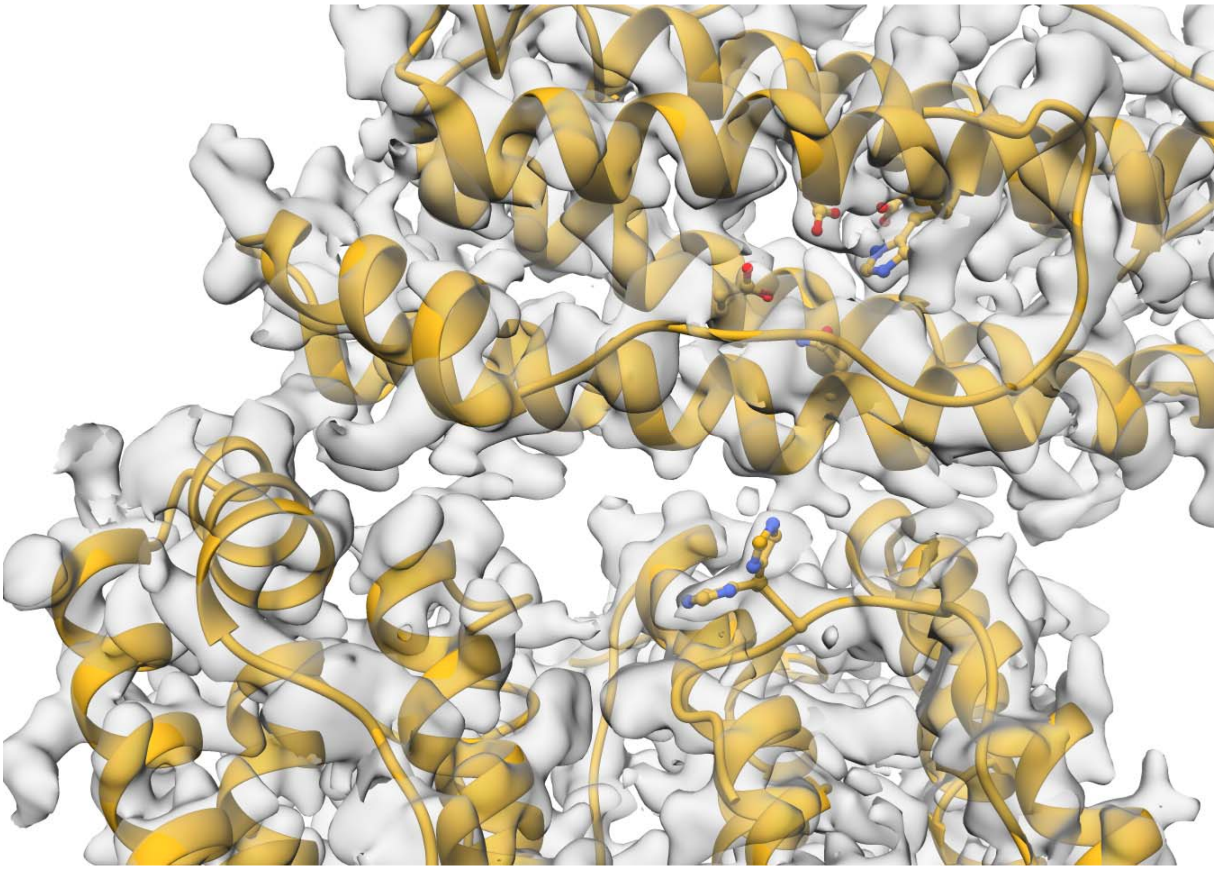

Figure S4 Cryo-EM map and *Mtb* BfrB model with the B-pore channel shown in the middle with a double conformation of His175. His175 as well as the residues at the ferroxidase sites are shown in ball-stick model.

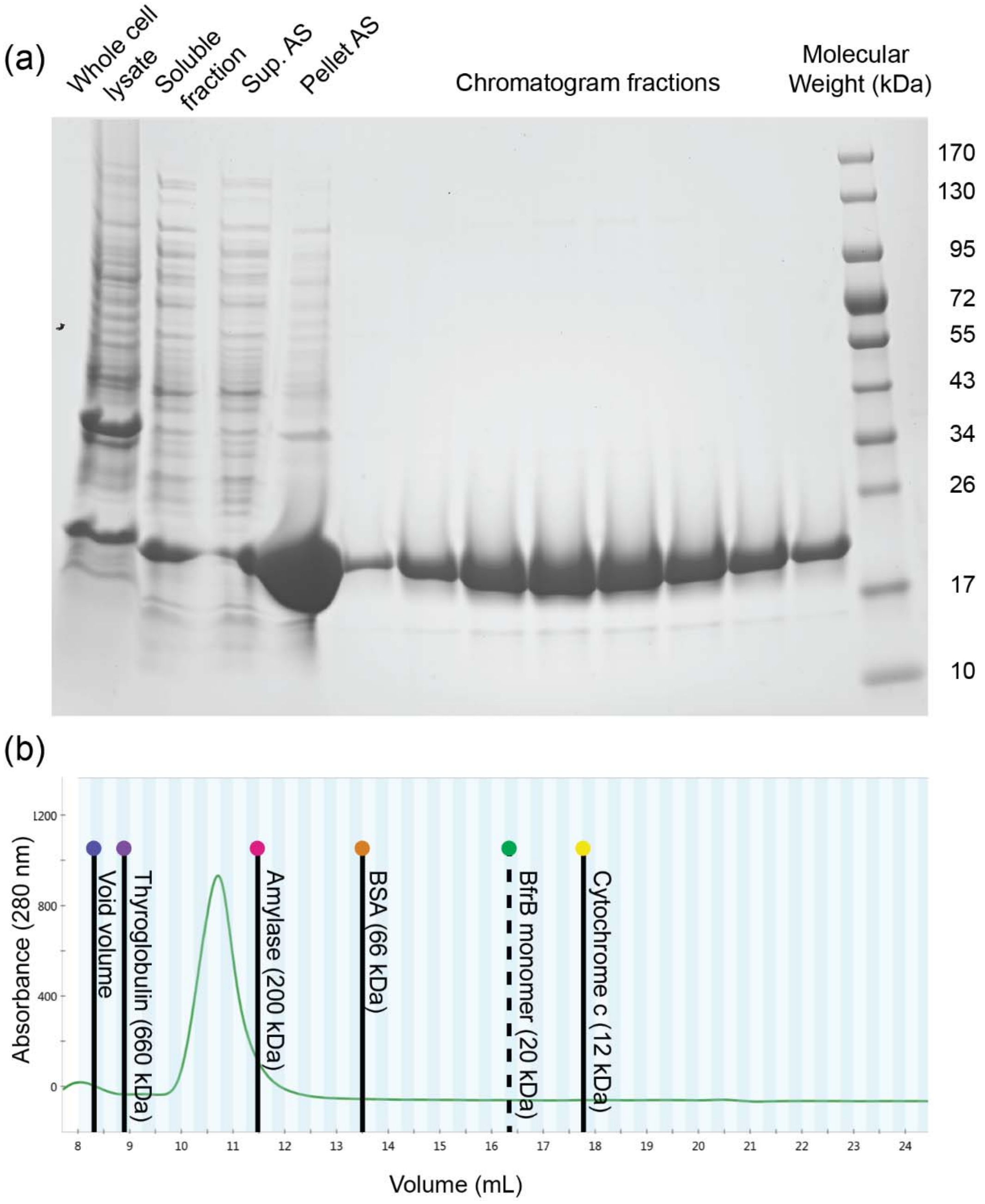

Figure S5 Purification of BfrB purification. (a) Denaturing gel electrophoresis of the different purification steps. Lysis of bacterial suspension (Whole cell lysate), removal of cellular debris by centrifugation (Soluble fraction), protein precipitation by ammonium sulfate (Supernatant AS and Pellet AS). Fractions of BfrB oligomer peak obtained from (b) size exclusion chromatography. Reference proteins used for column calibration positioned in their corresponding elution volume.

References

Kaur, S., Gomez-Blanco, J., Khalifa, A. A. Z., Adinarayanan, S., Sanchez-Garcia, R., Wrapp, D., McLellan, J. S., Bui, K. H. & Vargas, J. (2021). *Nature Communications* **12**, 1240.

Rosenthal, P. B. & Henderson, R. (2003). *J Mol Biol* **333**, 721-745.
